# Ameliorating Effect of Gemigliptin on Renal Injury in Murine Adriamycin-Induced Nephropathy

**DOI:** 10.1155/2017/7275109

**Published:** 2017-02-23

**Authors:** Da Rae Kim, Shin Yeong Lee, Jin Sug Kim, Yang Gyun Kim, Ju-Young Moon, Sang Ho Lee, Tae Won Lee, Chun Gyoo Ihm, Kyung Hwan Jeong

**Affiliations:** ^1^Department of Medicine, Graduate School, Kyung Hee University, Seoul, Republic of Korea; ^2^Division of Nephrology, Department of Internal Medicine, Kyung Hee University Hospital, Seoul, Republic of Korea; ^3^Department of Nephrology, Kyung Hee University School of Medicine, Seoul, Republic of Korea; ^4^Division of Nephrology, Department of Internal Medicine, Kyung Hee University Hospital at Gangdong, Seoul, Republic of Korea

## Abstract

*Background*. Previous studies have shown the antiapoptotic and anti-inflammatory potential of DPP-IV inhibitor in experimental models of renal injury. We tested whether DPP-IV inhibitor (gemigliptin) ameliorates renal injury by suppressing apoptosis, inflammation, and oxidative stress in mice with adriamycin nephropathy.* Methods*. Mice were treated with normal saline (control), gemigliptin (GM), adriamycin (ADR), or adriamycin combined with gemigliptin (ADR+GM). Apoptosis, inflammation, and oxidative stress were analyzed via western blotting, real-time PCR, light microscopy, and immunofluorescence.* Results*. In the ADR+GM group, urine albumin creatinine ratio decreased significantly compared with that in the ADR group on day 15. Glomerulosclerosis index and tubulointerstitial injury index in mice with adriamycin-induced nephropathy decreased after gemigliptin treatment. ADR group showed higher levels of apoptosis, inflammation, and oxidative stress-related molecules compared with the control group. The upregulation of these molecules was significantly reduced by gemigliptin. In the ADR group, the staining intensities of WT-1 and nephrin reduced, but these changes were ameliorated in the ADR+GM group.* Conclusion*. We demonstrated that gemigliptin ameliorates nephropathy by suppressing apoptosis, inflammation, and oxidative stress in mice administered adriamycin. Our data demonstrate that gemigliptin has renoprotective effects on adriamycin-induced nephropathy.

## 1. Introduction

Dipeptidyl peptidase IV (DPP-IV) is a multifunctional protein; its actions differ depending on the cell type and conditions. Among the several functions of DPP-IV, the modulating effect of incretin hormones on the release of insulin is well known. DPP-IV proteins also play a major role in the immune response and regulation of apoptosis [1, 2].

Recent studies have suggested the renoprotective effect of DPP-IV inhibitors. Min et al. investigated the effects of a DPP-IV inhibitor on renal fibrosis in a murine model of ureteral obstruction [3]. They found that mice treated with a DPP-IV inhibitor showed marked suppression of interstitial fibrosis and tubular atrophy by attenuating inflammation, oxidative stress, and apoptosis. Jung et al. also reported that a DPP-IV inhibitor reduced podocyte apoptosis in a diabetic mouse model without reducing hyperglycemia [4].

DPP-IV is expressed in the human liver, intestine, and kidney; in particular, DPP-IV is highly expressed in the apical brush border surface of proximal renal tubular cells [5]. However, it is unclear whether the inhibition of DPP-IV affects the pathogenesis of renal disease.

Adriamycin is an antineoplastic agent primarily metabolized by hepatocytes and is a well-known cause of cardiac toxicity in humans. Several previous studies reported that rats and mice exposed to adriamycin developed glomerulosclerosis with podocyte injury, tubulointerstitial inflammation, and fibrosis.

Gemigliptin (DPP-IV inhibitor, LG Life Sciences, Republic of Korea) was developed for the treatment of diabetes. Considering the previously demonstrated antiapoptotic, anti-inflammatory, and antioxidative potential of DPP-IV inhibitor in renal injury experiments [2–4], we supposed that gemigliptin may have a renoprotective effect in a murine model of adriamycin-induced nephropathy.

The objective of this study was to evaluate the renoprotective effect of gemigliptin with a focus on glomerulosclerosis with podocyte injury. We treated the mice with adriamycin-induced nephropathy with gemigliptin to determine whether it could inhibit oxidative stress and inflammation in adriamycin-induced renal disease and thus subsequent renal injury.

## 2. Methods

### 2.1. Animal Model and Experimental Design

The experimental protocol involved the use of an in vivo murine model of adriamycin-induced nephropathy over a 30-day study period. Assays were performed using inbred male BALB/c mice (22–25 g, aged 7 weeks) from the animal facility of Kyung Hee University Medical Center, Seoul, Korea. All animals were kept at a constant temperature (22 ± 2°C) under a 12-h light/dark cycle and had free access to a standard diet and distilled water. Approval was obtained from the local ethical animal committee before the initiation of the study. The animals were divided into four treatment groups. Group A was the control group (normal saline). Group B (gemigliptin, GM group) was fed with chow containing 300 mg/kg gemigliptin (LG Life Sciences, Republic of Korea). Group C (adriamycin, ADR group) was injected with 11.5 mg/kg of adriamycin (doxorubicin hydrochloride, Sigma, USA) and fed with standard chow. Group D (adriamycin combined with gemigliptin, ADR+GM group) was treated with adriamycin and fed with chow containing 300 mg/kg gemigliptin. All mice were sacrificed on day 30 after the injection. Urinary albumin levels were determined using the Bio-Rad protein assay (Bio-Rad, USA), and urinary creatinine levels were determined using the Parameter™ Creatinine kit (R&D systems, USA).

### 2.2. Analysis of DPP-IV Activity and GLP-1 Levels

DPP-IV activity in the plasma and kidney tissue was determined using a continuous spectrophotometric assay with the Gly-Pro-pNA substrate. Enzymatic activity was analyzed by measuring an increase in absorbance at >390 nm resulting from the cleavage of the Gly-Pro-pNA substrate by DPP-IV. A typical reaction solution consists of 40% plasma, 400 *μ*M Gly-Pro-pNA, and 50 mM HEPES buffer (pH 7.4) in a total volume of 100 *μ*L. The release of pNA was measured continuously for 15 min using a 96-well plate spectrophotometer, SpectraMax 340PC384 Microplate Reader (Molecular Devices, USA) at 30°C. Enzyme activity was defined as the slope (in mOD/min) from 4 to 14 min. The levels of the glucagon-like peptide-1 (GLP-1) receptor in the kidney tissue were determined via quantitative real-time PCR. Plasma active GLP-1 levels were determined using an ELISA kit (Millipore, USA).

### 2.3. Analysis of Renal Morphology

Renal morphology was analyzed using light microscopy. The kidney tissue was fixed in 10% neutral buffered formalin, embedded in paraffin, cut into 3 *μ*m sections, and stained with periodic-acid-Schiff (PAS). After PAS staining, the degree of glomerular sclerosis was assessed on a semiquantitative scale: grade 0, all glomeruli normal; grade 1, sclerotic area up to 25% (minimal sclerosis); grade 2, sclerotic area 25–50% (moderate sclerosis); grade 3, sclerotic area 50–75% (moderate-to-severe sclerosis); grade 4, sclerotic area 75–100% (severe sclerosis). One hundred glomeruli in each group were examined using that semiquantitative scale, as previously described [6]. The glomerulosclerosis index (GSI) was calculated using the following formula: GSI = [(1 ×* n*_1_) + (2 ×* n*_2_) + (3 ×* n*_3_) + (4 ×* n*_4_)]/(*n*_0_ +* n*_1_ +* n*_2_ +* n*_3_ +* n*_4_), where *n*_*x*_ is the number of glomeruli in each grade of glomerulosclerosis. Tubulointerstitial injury index (TII) was evaluated using the point-counting method, as previously described [7].

### 2.4. Immunofluorescence

To evaluate WT-1 levels, tissue sections mounted on slides were incubated with a primary antibody (WT-1, Santa Cruz Biotechnology, USA, rabbit polyclonal antibody, 1 : 50 dilution) at 4°C overnight. To determine the number of podocytes in glomeruli, cells positive for WT1 and DAPI were counted. At least 30 glomeruli were randomly examined in each section under 400x magnification and the average in each group was determined. Anti-nephrin antibody (Nephrin, Progen, Germany, Guinea pig polyclonal antibody, 1 : 50 dilution) was used to assess nephrin levels. To semiquantitatively analyze nephrin levels, scores were assigned to individual glomeruli as follows: score 4, continuous staining was strikingly disrupted and staining intensity decreased such that it covered 0–25% of the glomerular tuft area; score 3, 25–50%; score 2, 50–75%; score 1, 75–100%. The final score of each mouse was calculated using the following formula: *S*_nephrin_ = [(1 × *n*_1_)+(2 × *n*_2_)+(3 × *n*_3_)+(4 × *n*_4_)]/(*n*_0_ + *n*_1_ + *n*_2_ + *n*_3_ + *n*_4_), where *n*_*x*_ is the number of glomeruli.

### 2.5. Western Blotting

The expressions of Bax, Bcl-XL, inducible nitric oxide synthase (iNOS), and nitrotyrosine were determined by immunoblotting of the renal extracts. Tissue was lysed in 500 *μ*L of lysis buffer containing 1 : 100 protease inhibitor cocktail and centrifuged at 14,000 rpm for 30 min. Protein concentration in the supernatant was measured (Bio-Rad, USA) and 25 *μ*g of each sample was loaded on 8–15% SDS–PAGE gels and electrophoresed. The proteins were transferred to nitrocellulose sheets. After washing and blocking, the membranes were incubated at 4°C with the following primary antibodies: anti-nitrotyrosine (Santa Cruz Biotechnology, USA, 1 : 100), anti-iNOS (Cell Signaling Technology, USA, 1 : 1,000), and anti-Bax and anti-Bcl-XL (Cell Signaling Technology, USA, 1 : 1,000). The secondary antibody, horseradish peroxidase-conjugated goat anti-rabbit IgG (KPL, USA) or horseradish peroxidase-conjugated goat anti-mouse IgG (KPL, USA), was then added at room temperature (15–20°C) for 1 h. The blots were developed using a chemiluminescent reagent system (SuperSignal West Pico Chemiluminescent Substrate; Thermo Fisher, USA) and the images were recorded on film.

### 2.6. Quantitative Real-Time Polymerase Chain Reaction

Total RNA was isolated using TRIzol reagent (Invitrogen, USA) following the manufacturer's instructions. The integrity of the total RNA was examined using 1.1% agarose gel electrophoresis, and the purity was determined based on the ratio of A260/A280 using a spectrophotometer. Samples with OD values of 1.9–2.0 were used. First-strand cDNA was synthesized using RNA to cDNA EcoDry™ Premix (Oligo dT; Clontech, USA). The qPCR protocol was designed following the minimum guidelines for publication of quantitative real-time PCR experiments.

### 2.7. Data Analysis and Statistics

The data were used directly for stability calculations (StepOne Software, version 2.1) or relative quantitation (RQ) of amplified cDNA targets was determined using the cycle threshold (ΔΔCT) method. Genes with the lowest standard deviation (SD) and coefficient of variation (CV) values were considered the most stable. A *p* value < 0.05 was considered significant. SPSS Statistics version 19 (IBM, USA) was used for all statistical analyses. One-way analysis of variance with Scheffe's or Tukey's post hoc test was performed for comparison between more than two groups. An unpaired* t*-test was used for data comparison between two groups.

## 3. Results

### 3.1. Physical and Biochemical Parameters

The comparison of biochemical test results among the four groups is presented in Table 1. Body weight was found to be markedly lower in the ADR group than in the control group. Urine albumin creatinine ratio (UACR) significantly decreased in ADR+GM group on day 15 (ADR group: 402.20 ± 138.23 mg/g versus ADR+GM group: 133.70 ± 60.57 mg/g, *p* = 0.049). Serum glucose levels were not significantly different among the four groups (*p* = NS).

### 3.2. Effects of Gemigliptin on DPP-IV Activity and Active GLP-1 Levels

**Figure 1:**
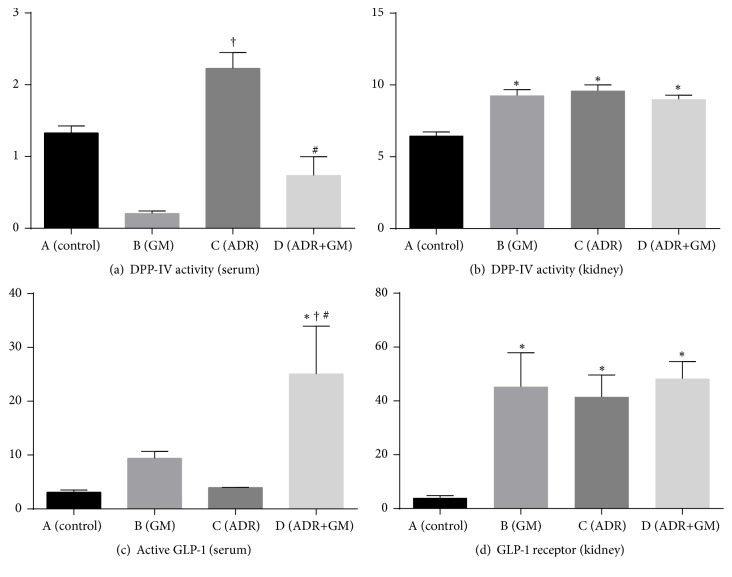
Effects of gemigliptin on DPP-IV activity and GLP-1 levels. ^*∗*^*p* < 0.05 compared with (A), ^†^*p* < 0.05 compared with (B), and ^#^*p* < 0.05 compared with (C).* p* values were obtained using the one-way analysis of variance test followed by Tukey's post hoc test. GM, gemigliptin; ADR, adriamycin; DPP-IV, dipeptidyl peptidase 4; GLP-1, glucagon-like peptide-1.

Serum DPP-IV activity significantly decreased in the ADR+GM group than in the ADR group (*p* < 0.01; Figure 1(a)). Renal DPP-IV activity in the control group was lower than that in the other groups, but there were no significant differences among the other three groups (Figure 1(b)). Serum active GLP-1 levels decreased in the ADR group and treatment with gemigliptin in the ADR group produced a statistically significant increment in serum active GLP-1 (Figure 1(c)). The level of the GLP-1 receptor in kidney tissue significantly increased in the GM, ADR, and ADR+GM group than in the control group, but there were no statistically significant differences among GM group, ADR group, and ADR+GM group (control group: 4.0 ± 0.9, GM group: 45.3 ± 12.6, ADR group: 41.5 ± 8.1, and ADR+GM group: 48.3 ± 6.3; Figure 1(d)).

### 3.3. Effects of Gemigliptin on Renal Structural Changes

**Figure 2:**
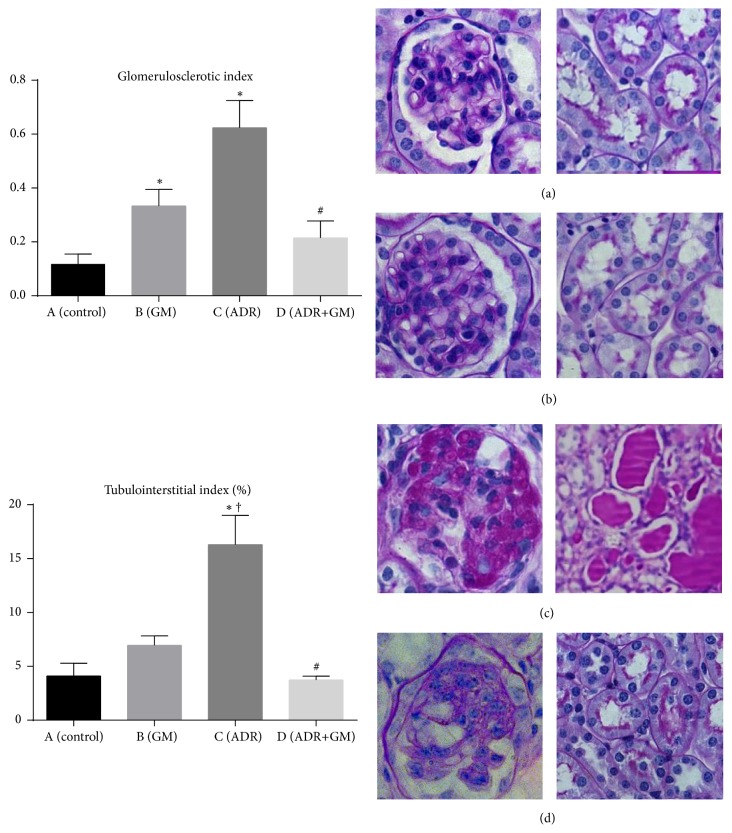
Glomerulosclerotic index and tubulointerstitial index (%). ^*∗*^*p* < 0.05 compared with (A), ^†^*p* < 0.05 compared with (B), and ^#^*p* < 0.05 compared with (C).* p* values were obtained using the one-way analysis of variance test followed by Tukey's post hoc test. Kidney samples (day 30) were sectioned and stained using periodic-acid Schiff (PAS). (A) Control group, (B) GM group, (C) global glomerular sclerosis and tubular injury in ADR group, and (D) focal segmental glomerular sclerosis in ADR+GM group. GSI, glomerular sclerotic index; GM, gemigliptin; ADR, adriamycin.

As observed via light microscopy, glomerular sclerosis, tubular atrophy, and interstitial fibrosis markedly increased and the proteinaceous material was observed in the tubular lumen of mice with adriamycin-induced nephropathy (Figure 2(c)). Compared with the ADR group, adriamycin-induced glomerular sclerosis and interstitial fibrosis significantly reduced in the ADR+GM group (Figure 2(d)). Mice in the ADR+GM group displayed significantly less glomerulosclerosis compared with mice in the ADR group ([GSI] ADR: 62.79% versus ADR+GM: 21.93%, *p* < 0.01). TII significantly increased in the ADR group but were found to be restored in the ADR+GM group ([TII] ADR: 16.29% versus ADR+GM: 3.74%, *p* = 0.012; Figure 2).

### 3.4. Effects of Gemigliptin on Oxidative Stress

**Figure 3:**
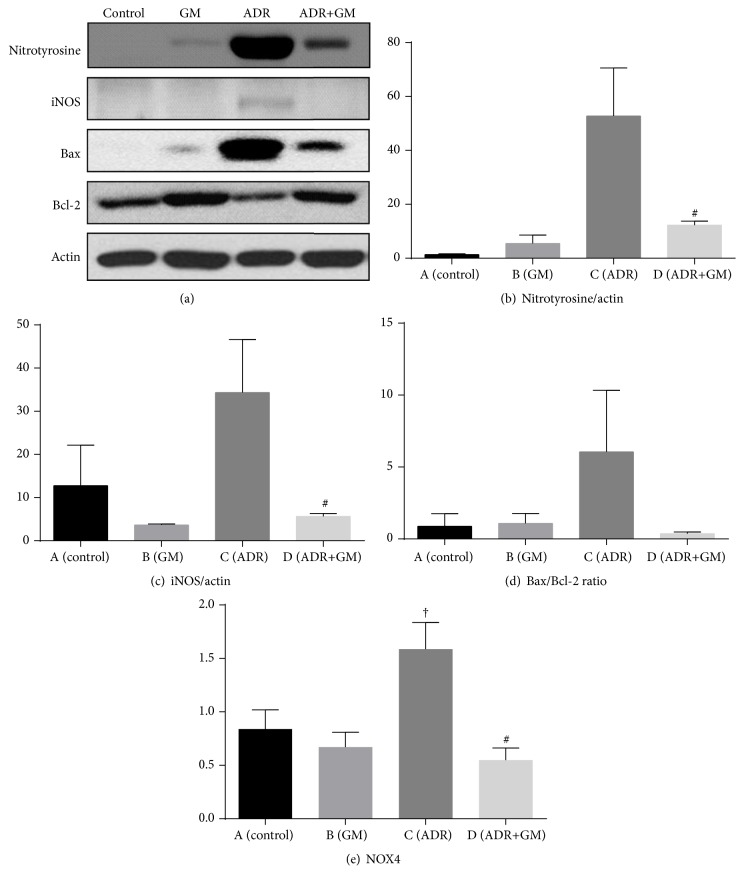
Results of western blot analysis of renal nitrotyrosine, iNOS, Bax/Bcl-2 ratio, and actin level (a, b, c, and d) and real-time PCR of NOX4 level of kidney tissue (e). ^*∗*^*p* < 0.05 compared with (A), ^†^*p* < 0.05 compared with (B), and ^#^*p* < 0.05 compared with (C). GM, gemigliptin; ADR, adriamycin; iNOS, inducible nitric oxide synthase; Bcl-2, B cell lymphoma 2; Bax, Bcl-2 associated X protein.

We examined the antioxidative mechanism of gemigliptin in the kidney of mice with adriamycin-induced nephropathy by measuring the levels of oxidative stress-related molecules. We found that nitrotyrosine, iNOS, and NADPH oxidase 4 (NOX4) were significantly upregulated in the ADR group but significantly downregulated by gemigliptin treatment (Figures 3(b), 3(c), and 3(e)).

### 3.5. Effects of Gemigliptin on Inflammation

**Figure 4:**
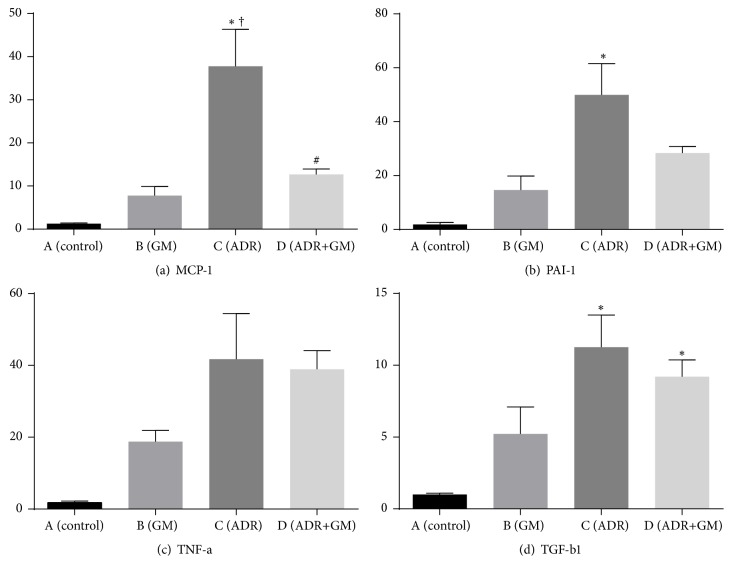
Real-time PCR of inflammation-related molecules (MCP-1, PAI-1, and TNF-*α*) and fibrotic cytokine (TGF-*β*1). TNF-*α* was not significantly different between the groups (*p* = 0.091).* p* values were obtained using the one-way analysis of variance test followed by Tukey's post hoc test. ^*∗*^*p* < 0.05 compared with (A), ^†^*p* < 0.05 compared with (B), and ^#^*p* < 0.05 compared with (C).

We examined the effects of gemigliptin on the levels of inflammatory cytokines in each group (Figure 4). Adriamycin significantly increased the levels of inflammatory cytokines, such as monocyte chemoattractant protein-1 (MCP-1) and plasminogen activator inhibitor-1 (PAI-1). Gemigliptin treatment in mice with adriamycin-induced nephropathy decreased the expression of these molecules. This suppression was statistically significant in the case of MCP-1 (Figure 4(a)). Although TNF-*α* tended to be higher in the ADR group and lower in the ADR+GM group, the difference in levels between the two groups was not significant (*p* = 0.091; Figure 4(d)).

### 3.6. Effects of Gemigliptin on Apoptosis and Fibrosis Markers

To evaluate the effects of gemigliptin on apoptosis-related molecules, we analyzed the expression of Bax and Bcl-2 in the kidney samples of each group. ADR significantly increased the expression of Bax and decreased the expression of Bcl-2 protein in the kidney. The increased Bax/Bcl-2 ratio, a marker of apoptosis, was effectively inhibited by treatment with gemigliptin (Figure 3(d)).

Though the molecular mechanisms underlying the role of TGF-*β*1 in apoptosis remain unclear, apoptosis is known to be associated with dysregulation of TGF-*β*1 activation and signaling. Therefore, we examined the effects of gemigliptin on TGF-*β*1 as a marker of fibrosis in each group. TGF-*β*1 levels significantly increased in the ADR group and marginally increased in the ADR+GM group (control group: 1.01 ± 0.15, ADR group: 11.25 ± 5.48, and ADR+GM group: 9.20 ± 3.52, *p* = 0.03; Figure 4(d)).

### 3.7. Effects of Gemigliptin on Podocyte Injury

**Figure 5:**
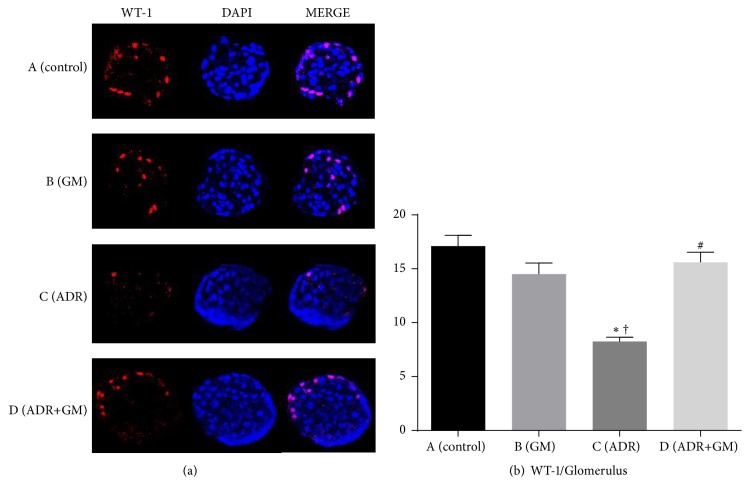
Effects of gemigliptin on adriamycin-induced podocyte loss (a); immunofluorescence stain for WT-1 (red) and DAPI (blue) and double-positive cells (pink) (b); mean podocyte number per glomerulus in each group is presented and shown for DAPI-positive and WT-1 positive cells; the asterisk indicates that the difference in mean podocyte number per glomerulus between groups is significant.* p* values are obtained using the one-way analysis of variance test followed by Tukey's post hoc test. ^*∗*^*p* < 0.05 compared with (A), ^†^*p* < 0.05 compared with (B), and ^#^*p* < 0.05 compared with (C). GM, gemigliptin; ADR, adriamycin; WT-1, Wilms tumor-1; DAPI, 4′,6-diamidino-2-phenylindole.

Many previous studies have shown that podocyte depletion leads to glomerulosclerosis [8]. To calculate the number of podocytes in glomeruli, the number of WT-1 and DAPI double-positive cells was counted. We found significantly decreased number of WT-1 positive nuclei in glomeruli in the ADR group compared with the control group (control group: 17.10 ± 1.02 versus ADR group: 8.27 ± 0.46, *p* < 0.05). In contrast, the number of nuclei was restored in mice with ADR-induced nephropathy that received gemigliptin (ADR group: 8.27 ± 0.46 versus ADR+GM group: 15.60 ± 1.12, *p* < 0.01; Figure 5(a)). In the ADR group, WT-1 staining intensity was reduced, but this change was inhibited in the ADR+GM group (ADR group: 8.27 ± 0.39 versus ADR+GM group: 15.60 ± 0.94, *p* < 0.0001; Figure 5(b)).

**Figure 6:**
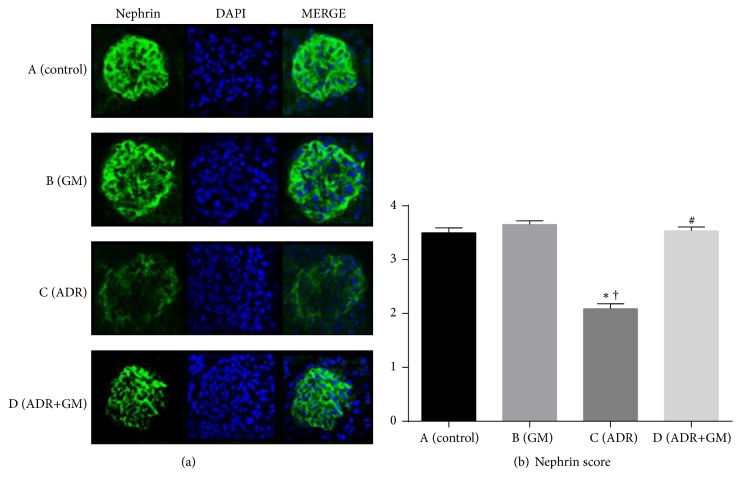
Effects of gemigliptin on adriamycin-induced downregulation of nephrin. (a) Immunofluorescence examination of nephrin expression. (b) Nephrin scores by semiquantitative analysis of immunofluorescence staining of nephrin (*p* < 0.0001).* p* values were obtained using the one-way analysis of variance test, followed by Tukey's post hoc test. ^*∗*^*p* < 0.05 compared with (A), ^†^*p* < 0.05 compared with (B), and ^#^*p* < 0.05 compared with (C). GM, gemigliptin; ADR, adriamycin; DAPI, 4′,6-diamidino-2-phenylindole.

The nephrin score was calculated as previously described [9] to semiquantitatively analyze nephrin levels. The nephrin score was reduced in the ADR group compared with the control group and was restored after treatment with gemigliptin in mice with adriamycin-induced nephropathy (ADR group: 3.54 versus ADR+GM group: 2.09, *p* = 0.04; Figure 6(b)).

## 4. Discussion

To the best of our knowledge, this is the first study to demonstrate that gemigliptin ameliorates renal injury and shows a renoprotective effect through antiapoptotic, antioxidative stress and anti-inflammatory mechanisms in a murine model of adriamycin-induced nephropathy. The three principal findings of our study are as follows: (1) gemigliptin demonstrates antioxidative, anti-inflammatory, and antiapoptotic effects without affecting glucose levels, (2) gemigliptin may ameliorate podocyte injury and show a renoprotective effect, and (3) gemigliptin prevents the progression of glomerulosclerosis and tubulointerstitial injury.

In 2006, the first DPP-IV inhibitor was approved for the treatment of diabetes. Glucagon increases blood glucose levels, and DPP-4 inhibitors reduce glucagon and blood glucose levels. The mechanism of DPP-4 inhibitors is to increase incretin levels, which inhibit glucagon release, which in turn increases insulin secretion and decreases blood glucose levels.

After several experimental and human studies have reported that DPP-IV inhibitors induce a reduction in the levels of proinflammatory cytokines, it has been gaining increasing attention for its role in inflammatory pathways [10, 11]. The exact mechanisms underlying this effect are unclear, but the involvement of mitogen-activated protein kinase (MAPK) and nuclear factor-kappa B (NF-kb) signaling pathways has been suggested by recent studies [2, 12]. Our findings showed that gemigliptin inhibits the expression of MCP-1. Many oxidative stress-related cytokines may be involved in the increase of reactive oxygen species (ROS) [13]. Both oxidative stress and inflammation are closely linked to renal injury. Many previous studies suggested that oxidative stress has a major role in the progression of fibrosis and end-stage renal disease. Nitrotyrosine is considered an indicator of reactive nitrogen species-induced stress, which has been reported to be associated with mortality in patients with acute kidney injury [14]. NOX4, which is ubiquitously expressed but shows particularly high expression in the kidney, was proposed to be a major source of ROS in diabetic nephropathy [15]. Our results are consistent with those of previous studies that reported that DPP-IV inhibitors show antioxidant and anti-inflammatory effects and are protective against apoptosis and fibrosis [3, 4].

Diabetic chronic kidney disease (DKD) is the most advanced area of research on DPP-IV inhibitors and renal injury. Recent studies have shown the benefits of gemigliptin in db/db mice without an influence on glycemic control [4]. However, diabetes treatment would impact the results from the analysis of the direct renal effects in the DKD model. A few studies have examined the effects of DPP-IV inhibitors on non-DKD models. Lim et al. suggested that a DPP-IV inhibitor protects against tacrolimus-induced renal injury through a reduction in oxidative stress and subsequent apoptosis [16]. Recently, Higashijima et al. [17] observed that a DPP-IV inhibitor reduced macrophage infiltration through the GLP-1 dependent signaling pathway in a rat anti-Thy-1 glomerulonephritis model. In our study, DPP-IV activity in the serum and kidney tissue decreased and activation of the GLP-1 receptor increased after the treatment with gemigliptin in mice with adriamycin-induced nephropathy; however, there were no statistically significant differences in blood glucose levels among the groups. Therefore, we are able to interpret the results of our study without the confounding factor of decreased blood glucose level.

Proteinuria is the principal manifestation of glomerular injury, and progression of proteinuria is a key pathogenic factor in renal dysfunction [18]. A reduced number of podocytes and alterations in slit-diaphragm proteins lead to the development of proteinuria [19]. Early events in the damaged podocyte are alterations of the slit diaphragm, reorganization of the foot process structure with the fusion of filtration slits, and apical displacement. If these changes persist, it can lead to progressive glomerular injuries and development of proteinuria. These early podocyte structural changes may be visible in more detail by using of electron microscopy [20].

Our data show that the attenuation of transcription of WT-1 and nephrin in adriamycin-induced nephropathy were restored by treatment with gemigliptin. In other words, we observed the restoration of podocyte loss and decreased slit-diaphragm proteins in glomerulosclerosis. WT-1 is expressed in podocytes and may play a key role in the maintenance of normal podocyte function. Thus, an adequate amount of WT-1 in podocytes represents normal podocyte maturity. The loss of renal podocytes is a contributing factor in the progression of chronic kidney disease. Nephrin which is known as a major structural component of the slit diaphragm is expressed on the surface of podocytes and is essential for the maintenance of normal glomerular permeability [21]. Previous studies investigated its role in the pathogenesis of proteinuric kidney diseases [22, 23]. Hauser et al. demonstrated that damage to endothelial cells may alter endothelial-podocyte interaction and induces nephrin loss, a main cause of proteinuria such as preeclampsia, hypertension, and diabetes [24]. Li et al. suggest that nephrin is critical for glomerular function and preservation of nephrin expression is a potential therapeutic approach in glomerular disease to mitigate podocyte loss and glomerular injury [25]. In our study, we demonstrated that gemigliptin ameliorates the attenuated expression of nephrin in mice with adriamycin-induced nephropathy.

In previous studies, it was reported that adriamycin injection led to a significant increase in the number of apoptotic podocytes followed by glomerulosclerosis in a rodent model [26, 27]. Furthermore, a DPP-IV inhibitor ameliorated diabetes-induced podocyte injury [4]. Our study suggested that the effects of gemigliptin on oxidative stress and apoptosis prevent podocyte loss due to renal injury.

Our study has several limitations. First, it is based on a murine model of adriamycin-induced nephropathy. Although this is highly reproducible and is widely used to evaluate renal injury, animal models are inherently limited in their ability to represent human chronic kidney diseases; nevertheless, our results provide a basis for future human studies. Second, we did not demonstrate the exact mechanism of action of gemigliptin on podocyte injury. We should have completed the study on alterations of podocytes by performing electronic microscopy to see podocyte effacement, but we could not do this. Instead, we demonstrate podocyte injury by IF staining of WT-1 and nephrin.

## 5. Conclusion

Our study clearly demonstrated that gemigliptin treatment leads to a reduction in apoptosis, inflammation, and oxidative stress in the murine model of adriamycin-induced nephropathy. Experimental and clinical studies are warranted for further insights.

## Figures and Tables

**Table 1:** Effects of adriamycin and gemigliptin on physical and biochemical parameters.

	A (control)	B (GM)	C (ADR)	D (ADR+GM)
Body weight (g)				
Day 0	24.70 ± 1.16	22.60 ± 0.71	25.30 ± 0.91^†^	24.90 ± 0.78^†#^
Day 8	26.20 ± 1.61	22.60 ± 0.53	26.40 ± 2.03^†^	23.00 ± 0.79^†#^
Day 15	26.80 ± 1.53	22.80 ± 0.91	23.40 ± 2.52	21.30 ± 1.58^*∗*^
Day 30	27.80 ± 1.04	23.20 ± 1.15	20.10 ± 9.61^*∗*^	20.90 ± 2.48^*∗*^
ΔBW	3.160 ± 0.29	0.60 ± 0.76	−4.70 ± 3.61^*∗*^	−4.00 ± 2.48^*∗*^
Urine albumin/creatinine (mg/g)				
Day 8	6.16 ± 0.55	7.29 ± 1.60	54.26 ± 42.05^*∗*^	79.55 ± 50.60
Day 15	11.66 ± 2.89	10.70 ± 1.04	402.20 ± 138.23^*∗*^	133.70 ± 60.57^*∗*#^
Day 30	8.33 ± 0.58	8.94 ± 1.83	223.90 ± 100.56^*∗*^	162.50 ± 104.57^*∗*^
Glucose (mg/dL)				
Day 30	108.44 ± 35.16	160.90 (*n* = 1)	124.99 ± 38.72	135.07 ± 10.55

^*∗*^ *p* < 0.05 compared with (A), ^†^*p* < 0.05 compared with (B), and ^#^*p* < 0.05 compared with (C).

Values for continuous variables; values are presented as mean ± standard deviation. *p* values were obtained using the one-way analysis of variance test followed by post hoc test. GM, gemigliptin; ADR, adriamycin; Alb, albumin; Cr, creatinine; ΔBW, difference in body weight.
